# Members of the *MYBMIXTA-like* transcription factors may orchestrate the initiation of fiber development in cotton seeds

**DOI:** 10.3389/fpls.2014.00179

**Published:** 2014-05-01

**Authors:** Frank Bedon, Lisa Ziolkowski, Sally A. Walford, Elizabeth S. Dennis, Danny J. Llewellyn

**Affiliations:** Commonwealth Scientific and Industrial Research Organisation, Plant Industry, Cotton BiotechnologyCanberra, ACT, Australia

**Keywords:** Gossypium, ovule, protoplast, transient assay, phylogeny, transcription, HDzip, fiber initiation

MYBMIXTA-like (MML) transcription factors form the subgroup 9 of R2R3-MYBs (Stracke et al., 2001) whose first characterized member was *MIXTA* from *Antirrhinum majus*. Various *MML* genes have been shown to be important regulators of epidermal cell differentiation in different plant species, including specifying cell shape in petals, vegetative trichome initiation and branching and seed fiber initiation (Martin et al., 2002; Machado et al., 2009; Walford et al., 2011). Indeed, the conical cells of petals look very much like young fibers shortly after they protrude from the epidermal surface of the cotton seed and begin to elongate, so it is likely there is some commonality in cellular regulation between the different tissue types. In tetraploid cotton, *Gossypium hirsutum* L. (*Gh*), the silencing or over-expression of two *MML* genes (*GhMYB25* and *GhMYB25Like)* expressed predominantly in the ovule epidermis during fiber initiation affect the initiation or timing of expansion of fiber initials (Machado et al., 2009; Walford et al., 2011). Based on silencing phenotypes and gene expression data, *GhMYB25Like* may be one of the most upstream genes in a regulatory cascade currently known to involve *GhMYB25*, *GhMYB109* (an R2R3 MYB from subgroup 15, Pu et al., 2008) and other types of transcription factors, such as the homeodomain leucine zipper (HDzip) factor *GhHD-1*, that may act in a protein complex with GhMYB25 (Zhang et al., 2010; Walford et al., 2012). The recent release of the genome sequence from the diploid *G. raimondii* (*Gr*), an extant species most closely related to the D-genome progenitor of tetraploid cotton, may help draw a more complete picture about the evolution of the *MML* gene subgroup in cotton and their apparent expansion and recruitment to specialized functions in epidermal seed fiber development.

## Genomic organization and phylogeny of cotton MMLs

**Figure 1:**
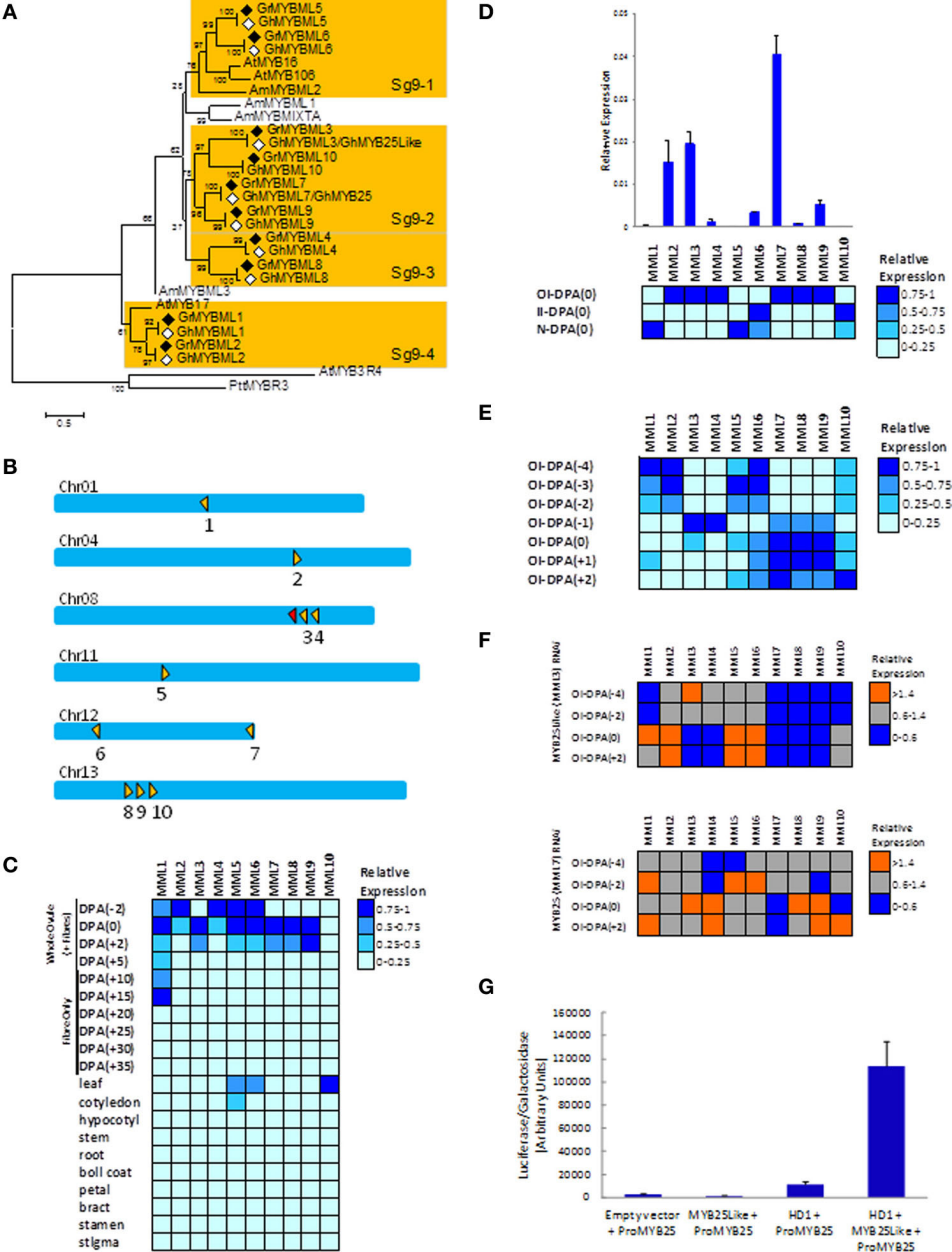
**Phylogenetic analysis, chromosomal location, gene expression of the cotton *MML* genes in wild type and transgenic *G. hirsutum*, and transactivation of the *GhMML3* promoter in cotton protoplasts**. **(A)** Phylogenetic analysis of the *G. raimondii* (Gr, filled diamonds) MML proteins and their putative *G. hirsutum* (Gh, empty diamonds) D-genome homologs. Four clades (Sg9-1 to 4) are indicated by shading. The rooted Neighbour–Joining tree was obtained in MEGA 5.0 with Clustal W alignment using the full length amino acid sequences (details in Supplementary data 1). *Arabidopsis thaliana* (At) MYB3R4 and *Populus tremula × P. Tremuloides* (Ptt) MYBR3 are R1R2R3-MYBs used as outgroups and the other AtMYB and *Anthirrinum majus* (Am) sequences are landmarks of Subgroup 9. **(B)** Schematic of the chromosomal distribution of the 10 *GrMML* genes indicated by the filled triangles, while the unfilled triangle represents a fragment of a *MML* gene. The directions of the triangle indicate the coding strand of the transcripts and the numbers under the triangle the particular *GrMML* gene. Adjacent triangles are tandemly arranged genes. (Chromosomes are only approximately to scale). The heat maps visualize the transcript level differences between the *G. hirsutum* homologues of the *GrMML* genes in: **(C)** cotton fibers, ovules and selected other plant organs, **(D)** three dissected tissues (OI, II and N) from wild type (*G. hirsutum*) ovules collected the day of anthesis [DPA(0)], **(E)** dissected OI of wild type ovules collected from 4 days before anthesis [DPA(−4)] to 2 days after anthesis [DPA(+2)], **(F)** OI dissected from *G. hirsutum* ovules silenced by RNAi for *GhMYB25Like* (ie., *MYBML3*) and *GhMYB25* (i.e., *MYBML7*) and their respective controls (ie., null segregant plants) collected at DPA(−4), (−2), (0), and (+2) **(F)**. Heat maps were made using Expander software based on gene expression relative to the cotton ubiquitin gene and normalized for each *MML* gene and separate experiment (details in Supplementary data 2). Primers used detect both the A- and D-genome homoeologues of each *MML* gene. **(G)** Transactivation assay of the *GhMML3/GhMYB25* promoter-Luciferase reporter by GhMYB25Like and/or GhHD-1 in cotton cotyledon protoplasts (details in Supplementary data 3).

The *Gr* genome contains over 200 R2R3 MYBs (Paterson et al., 2012), but 10 (*GrMML1-10*, Figure 1A) cluster with MIXTA, and all of these contain the signature protein motif AQWESARxxAExRLxRES previously indicated to be unique to subgroup 9 (Stracke et al., 2001). This number is considerably greater than the three *MML* genes found in Arabidopsis: *AtMYB16*, proposed to control the shape of petal epidermal cells (Baumann et al., 2007), *AtMYB17* a putative regulator of early inflorescence development and seed germination (Zhang et al., 2009) and *AtMYB106/NOK* a negative regulator of trichome branching (Jakoby et al., 2008). The *MML* factors are distinct from the *AtGL1*-like MYBs (*AtMYB0*, *AtMYB23*, *AtMYB66*) of subgroup 15, involved in trichome and root hair initiation and development, that are often suggested as the likely models for regulators of seed fiber development in cotton. Subgroup 15 in *Gr* appears to have only one member, Gorai.012G061800/GrMYB109 (Paterson et al., 2012) that is a homolog of GhMYB109 with a demonstrated role in fiber elongation rather than initiation (Pu et al., 2008). *Gr* has very close homologs of *GhMYB25* (Gorai.012G186500, *GrMML7*) and *GhMYB25Like* (Gorai.008G179600, *GrMML3*) as well as eight other novel *MML* genes, each with a pair of very close homologs in the A- and D-genomes of tetraploid *Gh* (Figure 1A, Supplementary data 1). The 10 MML proteins fall into 4 distinct clades supported by elevated bootstrap scores. One of these, Sg9-2, includes GrMML3/GhMYB25Like, GrMML7/GhMYB25, Gr/GhMML9 and 10, but no Arabidopsis or Antirrhinum MML MYBs (Figure 1A). In the *Gr* genome the 10 *MML* genes are distributed across 6 of the 13 diploid cotton chromosomes. However, on chromosomes Chr08 and Chr13 there are clusters of two (*GrMML3* and *4* and a fragment of another *MML* gene) and three genes (*GrMML8, 9*, and *10*), respectively, in tandem arrangements (Paterson et al., 2012; Figure 1B). This suggests that they may have evolved from gene duplications of ancestral *MML* genes and subsequently evolved new functions and in some cases different expression patterns.

## MML gene expression during the early events of fiber initiation and development

The 10 *MML* genes are nearly all expressed predominantly in early fiber development in whole ovules/seeds of tetraploid cotton from −2 to +2 day post anthesis (DPA) (Figure 1C) with little expression in other cotton tissues. A few (*GhMML5, 6, 10*) are also expressed in leaves and/or cotyledons which are rich in either hair or glandular trichomes that are structurally related to seed fibers and share some common regulators like *GhHD-1* (Walford et al., 2012). *MML* gene expression levels were also investigated using hand-microdissected tissues (Bedon et al., 2013) from whole ovules and young seeds to focus just on the fiber initiation process occurring in the epidermis. This circumvents the transcript dilution or confounding with expression from the other layers of ovule tissues. Six of the *GhMML* genes were preferentially expressed at 0 DPA in outer-integuments (OI; *GhMML2, 3, 4, 7, 8*, and *9*), two in inner-integument (II; *GhMML6* and *10*) and two in the nucellus (N; *GhMML1* and *5*) (heat map in Figure 1D). Based on their differences in relative expression levels in the OI, three groups can be classified as having; high (*GhMML2, 3* and *7*), low to medium (*GhMML 4, 6, 8*, and *9*), and very low (*GhMML1, 5*, and *10*) expression (chart in Figure 1D). Further expression analysis in the OI was performed from −4 to +2 DPA which covered the stages of ovule epidermal cell differentiation (−4 to −1 DPA), fiber initiation (i.e., ballooning of fiber initials above the epidermis surface at 0 DPA), and early fiber elongation (+1 to +2 DPA) (Figure 1E). There are four different patterns with some *GhMML* genes having their highest expression from −4 to −2 DPA (*GhMML1, 2, 5, 6*); genes peaking at −1 DPA (*GhMML3* and *4*); genes peaking at 0 to +1 DPA (*GhMML7, 8, 9*); and one member with highest expression at +2 DPA (*GhMML10*) (Figure 1E). These expression patterns support the specialization of different sets of *MML* genes for specific aspects of epidermal cell differentiation, although they may still have some roles in other tissues.

## MML regulatory cascades and interactions

To unravel the potential transcriptional networks among the different *GhMML* members their expression levels were assessed in dissected OI from transgenic tetraploid cotton silenced (through RNAi) for *GhMML3/GhMYB25Like* (Walford et al., 2011) or *GhMML7/GhMYB25* (Machado et al., 2009) compared to the transcript levels from the corresponding null segregant plants as controls (Figure 1F, Supplementary data 2). In the *GhMYB25Like* silenced plants Figure 1F (upper panel), the transcript level of *GhMYB25Like/GhMML3* was decreased at 0 and +2 DPA, as previously reported (Walford et al., 2011). Transcripts of *GhMML7/GhMYB25, GhMML8*, and *9* were completely abolished at 0 and +2 DPA, and *GhMML4* was decreased to a lesser extent, indicating that they may all be downstream of *GhMYB25-like*. A significant increase in *GhMML2* and *6* transcripts were observed at 0 and +2 DPA (Figure 1F), suggesting that they may be repressed by GhMYB25-like. A slight decrease in *GhMML1* at −4 DPA was also seen (see chart representation in Supplementary data 2). In the *GhMYB25* silenced plants (Figure 1F- lower panel), the transcript levels of *GhMML7/GhMYB25* was decreased at 0 and +2 DPA as previously reported by Machado et al. (2009). Transcript levels of *GhMML3/GhMYB25Like, GhMML4* and *8*, and to a lesser extent *GhMML9*, were increased at 0 DPA (Supplementary data 2), suggesting there may be some feedback regulation of *GhMYB25Like* by GhMYB25. The direct requirement of GhMYB25Like/GhMML3 for expression of *GhMML7/GhMYB25* was assessed using a transient assay in cotton protoplasts (Figure 1G, Supplementary data 3). Transactivation of the *GhMYB25* promoter, fused to the *luciferase* reporter, did occur in the presence of GhMYB25Like, but only when co-expressed with the HDzip factor GhHD-1, recently shown to be involved in fiber initiation (Walford et al., 2012), so the two may be involved in a complex to activate *GhMYB25*.

## Conclusion

The MYBMIXTA-like subgroup appears to have expanded in cotton compared to non-fiber plants, probably following a cotton specific triplication as reported in Paterson et al. (2012), thus contributing to the evolution of seed fiber. The spatio-temporal expression study of the different *GhMML* genes reported here distinguishes them according to the plant organs and/or ovule tissues in which they are expressed and the timing of their expression during the early stages of seed fiber development. The newly described *GhMML2* is one of the more highly expressed *MML* genes in the OI along with *GhMML7/GhMYB25* and *GhMML3/GhMYB25Like* which have already been shown to be involved in seed fiber initiation (Machado et al., 2009; Walford et al., 2011), but *GhMML2* shows a different pattern of expression with highest transcript accumulation before the fiber initiation step. *GhMML2*, together with *GhMML6*, transcripts are increased rather than decreased at 0 and +2 DPA in *GhMYB25Like* RNAi plants, suggesting a molecular function different to *GhMML7*, *8*, and *9* that are down-regulated in this transgenic background. The differences in *GhMML* expression in OI in the two *MMLs* silenced plants highlights a possible hierarchical network between MMLs; with MML3/GhMYB25-like activating the transcription of *MML7/GhMYB25*, *MML8*, and *MML9* and perhaps being involved in the repression of *MML2* and *MML6* at 0 and +2 DPA as the fibers begin to expand and elongate. MML7/GhMYB25 might be involved in a feedback loop to transcriptionally activate *MML3*, *4*, *8*, and *9* at 0 DPA. We were able to show that MML3/GhMYB25Like is able to transactivate the *MML7/MYB25* promoter, but only when associated with GhHD-1. Such results suggest the presence of a transcriptional complexes, involving MML(s) and GhHD-1, necessary for fiber development that are similar to the sorts of complexes seen in Arabidopsis trichome development (Ishida et al., 2008), although clearly involving different factors. Here, we identified some potential new players in fiber development that belong to the same phylogenetic group of MYBs and suggest a very subtle networking involving both protein-protein and protein-DNA interactions. The next steps involve the functional characterization of these novel cotton *MML* genes by silencing and over-expression in transgenic cotton and the study of their interactions in both cotton protoplasts and yeast to confirm their roles in generating the fibers of commercially important cotton species.

## Author contributions

Phylogeny analysis (Figure 1A): Frank Bedon. Genomic organization of *MMLs* (Figure 1B): Danny J. Llewellyn. Gene expression studies (Figures 1D–F): Frank Bedon and Lisa Ziolkowski, (Figure 1C): Sally A. Walford. Transient assays in protoplast (Figure 1G): Frank Bedon and Lisa Ziolkowski. Wrote the paper: Frank Bedon, Sally A. Walford, Elizabeth S. Dennis and Danny J. Llewellyn.

### Conflict of interest statement

The authors declare that the research was conducted in the absence of any commercial or financial relationships that could be construed as a potential conflict of interest.

## Supplementary material

Supplementary data 1 **Sequence information**.

Supplementary data 2 **Gene expression data**.

Supplementary data 3 **Transient assays**.
