# Supplementary material for: Members of the MYBMIXTA-like transcription factors may orchestrate the initiation of fiber development in cotton seeds
Source: Front Plant Sci. 2014 May 1;5:179. doi: 10.3389/fpls.2014.00179 (PMC4028877; doi:10.3389/fpls.2014.00179)
Supplement: Supplementary data 1 — Sequence information. [file DataSheet1.DOC]

**Supplementary data 1 (for Figure 1A). Phylogenetic analysis of the *G. raimondii* MML proteins and their *G. hirsutum* homologues belonging to the MIXTA clade (subgroup 9).**

The rooted Neighbour–Joining tree was constructed by MEGA 5.0.5 software using the full amino acid sequences of the MYBs with the Jones–Taylor–Thornton substitution model (Gamma parameter of 1.0 and pairwise gap deletion). Clustal W sequence alignments were obtained with the MEGA 5.2.1 software (Tamura *et al.*, 2011), with parameters set as follows: gap opening penalty/gap extension penalty of 25/1 for pairwise alignment and 5/1 for multiple alignment by using the blosum protein weight matrix for amino acid sequences. The analysis involved 29 amino acid sequences. All ambiguous positions were removed for each sequence pair. There were a total of 1018 positions in the final dataset. The bootstrap consensus tree was inferred from 1000 replicates. *Gossypium raimondii* sequences identified in this study are shown with filled lozenges in Figure 1A while *G. hirsutum* are in empty lozenges; landmarks from this subgroup 9 are from *Arabidopsis thaliana* (At) and *Antirrhinum majus* (Am)*.* AtMYB3R4 and PttMYBR3 (*Populus tremuloides x P. tremula*) are the outgroups. Bootstrap values are shown at branch nodes. Scale bar indicates the evolutionary distance as a percentage estimating the number of amino acid substitution per site.

**Tamura K., Peterson D., Peterson N., Stecher G., Nei M., and Kumar S.** (2011). MEGA5: Molecular Evolutionary Genetics Analysis using Maximum Likelihood, Evolutionary Distance, and Maximum Parsimony Methods. Mol. Biol. Evol. **10***,* 2731-3.

The primers pairs used to PCR amplify the full coding sequences for the *G. raimondii* homologues in *G. hirsutum* have been designed based on the *G. raimondii* genome sequence (Paterson et al., 2012) with the Primer3 software, and are the following:

| Primers | Sequence 5’-3’ | T° annealing | Length |
| --- | --- | --- | --- |
| GhMML1-FOWARD | ATGGGGAGGAGGCCATGTTGT | 55 | *21* |
| GhMML1-REVERSE | TCAACATTGCGGGGGTGTAGC | 55 | *21* |
| GhMML2-FOWARD | ATGGGGAGGACACCATGTTGTG | 55 | *22* |
| GhMML2-REVERSE | TCAACAATTTTCCAAGAAACTCATGTCATTG | 55 | *31* |
| GhMML4-FOWARD | ATGCTAAGTAAAATGCGGCCACCG | 55 | *24* |
| GhMML4-REVERSE | TTATCCCAACACAGGCGAACCACA | 55 | *24* |
| GhMML5-FOWARD | ATGGGGAGATCACCTTGCTGTGA | 55 | *23* |
| GhMML5-REVERSE | TTAGAACATTGGTGAGCCCGATGG | 55 | *24* |
| GhMML6-FOWARD | ATGGGGAGATCACCTTGCTGTGA | 55 | *23* |
| GhMML6-REVERSE | TTAGAACATTGGTGAATCTGATGGGGAA | 55 | *28* |
| GhMML8-FOWARD | AAAAATGGGGTGGCGGCT | 50 | *18* |
| GhMML8-REVERSE | TCATTTCATGACATTATGATCATATAAC | 50 | *28* |
| GhMML9-FOWARD | ATGGGACGGTCTCCATGCTG | 55 | *20* |
| GhMML9-REVERSE | TTAACTTACCAAATTAAGGATGTTATTCCAG | 55 | *31* |
| GhMML10-FOWARD | ATGGCTGATTGCCGGGAGAAGAT | 55 | *23* |
| GhMML10-REVERSE | TTAAACGGTTATAGACTCCTCGTTGTCA | 55 | *28* |

The amplified PCR products were gel extracted, ligated to pGEMT and then sequenced.

The 29 aminoacid sequences (accession numbers or G. raimondii gene models in brackets) (*G. hirsutum* sequences are for the putative D-genome homologs) used to make the phylogenetic tree are:

>GrMYBML1(Gorai.001G169700.1)

MGRRPCCERKGLKKGPWGPEEDEILINYINKHGHGSWRSLPKLSALWKFLFCRVSPLQPIKGLISTLTGLRRCGKSCRLRWTNYLRPDIKRGPFTLDEEKLVIQLHAILGNRWAAIASQLPGRTDNEIKNLWNTHLKKRLLCMGVDPLTHEPFTSGGPTTRPRSSPATRHMAQWESARLEAEARLSKESLFFNSPPTSVKPDPDFFLRLWNSEVGESFRKLNGEAKADCRSPISQASSSTKCGSVSGVTIDVGPIAAGSSTPKSSRTEDPIMFDASFSSSSNESEDSSDTALQLLLDFPINNDMSFLDDVDTYATPPQC

>GhMYBML1

MGRRPCCERKGLKKGPWGPEEDEILINYINKHGHGSWRSLPKLSGLSRCGKSCRLRWTNYLRPDIKRGPFTLDEEKLVIQLHAILGNRWAAIASQLPGRTDNEIKNLWNTHLKKRLLCMGVDPLTHEPSTSGGPTTRPRSSPATRHMAQWESARLEAEARLSKESLFFNSPPTSVKPDPDFFLRLWNSEVGESFRKLNGEAKADCRSPISQASSSTKCGSVSGVTIDVGPIAAGSSTPKSSRTEDPIMFDASFSSSSNESEDSSDTALQLLLDFPINNDMSFLDDVDTYATPPQC

>GrMYBML2(Gorai.004G157600.1)

MGRTPCCDKKGLKKGPWAPEEDEILTNYIKKHGHGSWRSLPKLAGLLRCGKSCRLRWTNYLRPDIKRGPFTLEEEKLVIQLHAILGNRWAAIAAQLPRRTDNEIKNLWNTHLKKRLLCMGLDPQTHKPFTPCGGPTVAAPTSPATRHMAQWESARLEAEARLSKESLQTNSTPIAKPDSDHFLRLWNSEVGESFRKINTEYKTVYPCPSPISQTSSSTKCGSVSAVTMDVCPNIAGSLNPASNPIEETECKSFIKSCIEEPSDSSCSSESEDSSDTALQLLLDFPINNDMSFLENC

>GhMYBML2

MGRTPCCDKKGLKKGPWAPEEDEILTNYIKKRGHGSWRSLPKLAGLLRCGKSCRLRWTNYLRPDIKRGPFTLEEEKLVIQLHAILGNRWAAIAAQLPRRTDNEIKNLWNTHLKKRLLCMGLDPQTHKPFTPCGGPTVAAPTSPATRHMAQWESARLEAEARLSKESLQTNSPPIAKPDSDHFLRLWNSEVGESFRKINTEYKTVYPCPSPISQTSSSTKCGSVSAVTMDVCPNIAGSLNPASNPIEETECKSFIKSCIEEPSDSSCSSESEDSSDTALQLLLDFPINNDMSFLENC

>GrMYBML3(Gorai.008G179600.1)

MQQSPCSDKVGLKKGPWTPEEDQKLLSYIQEHGGGSWRGLPAKAGLQRCGKSCRLRWINYLRPDIKRGKFSSQEERTIIQLHALLGNRWSAIAAHLPKRTDNEIKNYWNTQLKKRLTKIGIDPATHRPKTDTLGSTPKDAANLSHMAQWESARLEAEARLVRESKRVSNPPQNQFRFTSSSAPPLVSKIDVGLAHATKPQCLDVLKAWQRVVTGLFTFNTDNLQSPTSTSSFTENRLPISSVGFIDSFLGNSNNSCCGNNWECVEKSSQVAELQERLDNSMGLHDILDFSSDDVWFQGSYRAENMMEGYSDTLMVCDSGDHQKSLSMEPRQNFNVGTSNASSFEENKNYWNNILNFANASPSGSSVF

>GhMYBML3/GhMYB25Like(ADZ98877)

MQQSPCSDKVVLKKGPWTPEEDQKLLSYIQEHGGGSWRGLPAKAGLQRCGKSCRLRWINYLRPDIKRGKFSSQEERTIIQLHALLGNRWSAIAAHLPKRTDNEIKNYWNTQLKKRLTKIGIDPATHRPKTDTLGSTPKDVANLSHMAQWESARLEAEARLVRESKRVSNPPQNQFRFTSSSAPPLVNKIDVGLAHATKPQCLDVLKAWQRVVTGLFTFNTDNLQSPTSTSSFTENRLPISSVGFIDSFVGNSNNSCCGNNWECVEKSSQVAELQERLDNSMGLHDILDFSSEDVWFQGSYRAENMMEGYSDTLMVCDSGDHQKSLSMEPRQNFNVGTSNASSFEENKNYWNNILNFANASPSGSSVF

>GrMYBML4(Gorai.008G179800.1)

MLSKMRPPSPNRKKEVRLKRGPWTAEEDKLLTAYIQKHGYGSWGSLPHKAGLERCGKSCRLRWINYLRPDIKRGKFSLEEEQTIIQLHAFLGNRWSAIAAHLPKRTDNEIKNHWNTHLKKRLIKMGIDPMTHKPSTSPSPKNGSNLSHMTQWESARLQAEARLVRESKQVVPNLTTRPTRRSQLTRSSPRCLDVLKAWQGIVAGMFVFSTQDPRSLTTSTLRFPGWVEAEEWRGQGKKGSSDADDAWFEEDSVILHSLPIANIMEGLSDAFILNSWMGVDKSTDENTVMENGNCWDSVLNFLNSSPCGSPVLG

>GhMYBML4

MLSKMRPPSPNRKKEVRLKRGPWTAEEDKLLTAYIQKHGYGSWGSLPHKAGLERCGKSCRLRWINYLRPYIKRGKFSLEEEQTIIQLHAFLGNRWSAIAAHLPKRTDNEIKNHWNTHLKKRLIKMGIDPMTHKPSTTPSPKNGSNLSHMAQWESARLQAEARLVRESKQVVPNLTTCPTRRSQLTRSSPRCLDILKAWQGVVAGMFVFSTQDPRSLTTSTLRFPSAGWGEAEEWRGQGMKGSSDADDAWFEEDSVILHSLPIENIMEGLSDAFILNSWMGVDKSTDENIVKENGNCWDSVLNLLSSTPCGSPVLG

>GrMYBML5(Gorai.011G122800.1)

MGRSPCCDKVGLKKGPWTPEEDQKLLAYIEEHGHGSWRALPVKAGLQRCGKSCRLRWTNYLRPDIKRGKFSMQEEQTIIQLHALLGNRWSAIATHLPKRTDNEIKNYWNTHLKKRLAKLGIDPITHKPKSDALLSTDAQSKSAANLSHMAQWESARLEAEARLVRESKLRSHSHSFQHRLTRPPTAAFASSAGRLVNKTAWNSTAGWSKSSEVNNGVVNNGFGDLESPKSTLTSSENGVGLSSMGMPDFVGTASASSEIKQEGEQEWKGFGSSTNLAMENGFNDIGNAMEDGFINLLLNDSTDPSLSDSGKESDGNSGDGTASDDHYEDNKNYWNSILDLVNSSPSGSPMF

>GhMYBML5

MGRSPCCDKVGLKKGPWTPEEDQKLLAYIEEHGHGSWRALPAKAGLQRCGKSCRLRWTNYLRPDIKRGKFSMQEEQTIIQLHALLGNRWSAIATHLPKRTDNEIKNYWNTHLKKRLAKLGIDPITHKPKSDALLSIDSQSKSAANLSHMAQWESARLEAEARLVRESKLRSHSHSFQHRLTRPPTAAFASSAGELVNKTAWNSTAGWSKSSEVNNGVVNNGFGDLESPKSTLTSSENGVGLSSMGMPDFVGTASASSQIKQEGEQEWKGFGSSTNLAMENGFNDIGNAMEDGFINLLLNDSTDPSLSDSGKESDGNSGDGIARDDHYEDNKNYWNSILDLVNSSPSGSPMF

>GrMYBML6(Gorai.012G052500.1)

MGRSPCCDKVGLKKGPWTPEEDQKLLAYIEEHGRGSWRSLPAKAGLQRCGKSCRLRWTNYLRPDIKRGKFSLQEEQTIIQLHALLGNRWSAIATHLPKRTDNEIKNYWNTHLKKRLAKMGIDPITHKPKNDALLSTTDGQSKKAANLSHMAQWESARLEAEARLVRESKIRSHSLQHHHHFNPPAFTLESPTSTLSVSENAPPIITGLGVSPMPMIEFVGTTSGSSETAGIVKEEGEQEWKELGSSSNLADYKEGMGNSLSSFTSSLQDMTISIEGGWTPESLRPNNINVNNVGNIMEEGFTNLLLNDSFNRSLSDSGKESDENSGGSGDGSDYYQDNKNYWNSILNLVNSSPSDSPMF

>GhMYBML6

MGRSPCCDKVGLKKGPWTPEEDQKLLAYIEEHGRGSWRSLPAKAGLQRCGKSCRLRWTNYLRPDIKRGKFSLQEEQTIIQLHALLGNRWSAIATHLPKRTDNEIKNYWNTHLKKRLAKMGIDPITHKPKNDALLSTTDGQSKKAANLSHMAQWESARLEAEARLVRESKIRSHSLQHHHHFNPPAFTLESPTSTLSVSENAPPIITGLGVSPMPMIEFVGTTSGSSETAGIVKEEGEQEWKELGSSSNLADYKEGMGNSLSSFTSSLQDMTISIEGGWTPESLRPNNINVNNVGNIMEEGFTNLLLNDSVNRSLSDSGKESDENSGGSGDGSDYYQDNKNYWNSILNLVNSSPSDSPMF

>GrMYBML7(Gorai.012G186500.1)

MGRSPCCEKVGLKKGPWTPEEDQKLLAYIEQHGHGSWRALPLKAGLQRCGKSCRLRWINYLRPDIKRGKFSLQEEQTIIQLHALLGNRWSAIATHLPKRTDNEIKNYWNTHLKKRLTKMGIDPVTHKPKTNALGSTTGNPKDAANLSHMAQWESARLEAEARLVRESKLVPSNPPQSNHFTAVAPSPTPATRPQCLDVLKAWQGVVCGLFTLNMDNNNLQSPTSTLNFMENTTTLPMSSSSVNGMFNENFGWNSSFNPCESGDILKVEYGSDQIPELKERLDHPMELHEMDCSSEGTWFQELFGFNGL

>GhMYBML7/GhMYB25(ACJ07153)

MGRSPCCEKVGLKKGPWTPEEDQKLLAYIEQHGHGSWRALPLKAGLQRCGKSCRLRWINYLRPDIKRGKFSLQEEQTIIQLHALLGNRWSAIATHLPKRTDNEIKNYWNTHLKKRLTKMGIDPVTHKPKTDALGSTTGNPIDAANLSHMAQWESARLEAEARLVRESKLVPSNPPQSNHFTAVAPSPTPATRPQCLDVLKAWQGVVCGLFTFNMDNNNLQSPTSTLNFMENTTTLPMSSSSSVNGMFNENFGWNSSINPCESGDILKVEYGSDQIPELKERLDHPMELHEMDCSSEGTWFQELFGFNGL

>GrMYBML8(Gorai.013G088200.1)

MGWRLPFNGHKEVGLKRGPWTAEEDQILMAYIQQHGHGNWRALPEKAGLKRCGKSCRLRWINYLRPDIKRGKFSLQEEHTIIQLHALLGNRWSAMAAHLPKRTDNEIKNYWNTHLKKRLIKLGVDPMTHKPRTDASSFPSGSNLTHMAQWESARLEAEARLVRDSKQVIPNPIHPKNHLTLNHIQLRPRCLDVLKAWQGVVAGMFAFPTQDLGSPTSTLRFPAIGLNATSYTDGEMGFDDSLKCIENSNQMKEIEETITDGCIDEWFEDSFRVGNYENVPMAVGSSNCCDSVFDLVNSSPYGLSMLYDHNVMK

>GhMYBML8

MGWRLPLNGHKEVGLKRGPWTAEEDQILMAYIQQRGHGNWRALPEKAGLKRCGKSCRLRWINYLRPDIKRGKFNLQEEHTIIQLHALLGNRWSAMAAHLPKRTDNEIKNYWNTHLKKRLIKLGVDPMTHKPRTDAASFPSGSNLTHMAQWESARLEAEARLVRGSKQVVPNPIHPKNHLTLNHSQLRPRCLDVLQAWQGVVAGMFAFPTQDLGSPTSTLRLPAIGLNATSYTDCEMGFDDSLKYIENSNQMKEIEETITDGCIDEWFEDSFRVGNYESVPMAVDGSNCCGSVFDLVNSSPYGLSMLYDHNVMK

>GrMYBML9(Gorai.013G088300.1)

MGRSPCCDKVGLKKGPWTPEEDQKLLAYIEQHGHGSWRALPAKAGLQRCGKSCRLRWINYLRPDIKRGKFSLQEEQTIIQLHALLGNRWSGIAAHLPKRTDNEIKNYWNTHLKKRLNKMGIDPVTHKPKTNALGSATGNPKDAATLSHMAQWESARLEAEARLVRESKLVRFSSSSSSSSSAPQHTSNPVMTPPATRPQCLDVLKAWQGLVTGLFTFNNTTDNLQSPTSTLNFVENTNTLANGLINDNSMELHEMGAWFRQDSSYRAVENMNMEDYSDMMVWESGDHQQCSSMAAPAENLNETSYGNSSSSSSSSSSLEETRNYWNNILNLVS

>GhMYBML9

MGRSPCCDKVGLKKGPWTPEEDQKLLAYIEQHGHGSWRALPAKAGLQRCGKSCRLRWINYLRPDIKRGKFSLQEEQTIIQLHALLGNRWSGIAAHLPKRTDNEIKNYWNTHLKKRLNKMGIDPVTQKPKTNALGSATGNPKDAATLSHMAQWESARLEAEARLVRESKFVGSSSSSSSSSSSSAPQHTSNTVMTPPATRPQCLDVLKAWQGLVTGLFTFNNTTDNLQSPTSTLNFVENTNTLANGLINENSMELHEMGAWFRQDSSYRAVENMNMEDYSDMMVWESGDHQQWSSMAAPAENLNETSYGNSSSSSSSLEENRNYWNNILNLVS

>GrMYBML10(Gorai.013G088400.1)

MADCREKMGLKKGPWTPDEDQKLLAYIEEHGLGNWRTFPEKAGLQRCGKSCRLRWINYLRPDLKRGKFSLQEEQTIIQLHAFLGNRWSTIAAHLPNRTDNEIKNYWNTHVKKRFTKMGIDPTTHKPKSNHVVSPTGRTTLNHMAQWESARLEAEARLVKDSKNLPSSSSRPSPYQKSCNKGSKSQCLDVVKAWQSVVAGMFATSTNNSNRIIFGPDQSSGNYELDSIIPIGGNVEDELMVGNDRSKCQVPELNERFDNYMSLHDTTHLWAAPIAENDVVEGFPDFLVHDFDYQIDNEESITV

>GhMYBML10

MADCREKMGLKKGPWTPDEDQKLLAYIEEHGLGNWRTLPEKAGLQRCGKSCRLRWINYLRPDLKRGKFSLQEEQTIIQLHAFLGNRWSTIAAHLPNRTDNEIKNYWNTHVKKRFTKMGIDPTTHKPKSNHVVSPTGRTTLNHMAQWESARLEAEARLVKDSKNLPSSSSRPSPYQKSCNKGSKSQCLDVVKAWQSVVAGMFATSTNNSNRIIFGPDQSSGNYELDSIIPIGGNVEDELMVGNDRSKCQVPELNERFDNYMSLHDTTHLWAAPIAENDVVEGFPDFLVHDFDYQIDNEESITV

>AtMYB16(NP_197035)

MGRSPCCDKLGLKKGPWTPEEDQKLLAYIEEHGHGSWRSLPEKAGLHRCGKSCRLRWTNYLRPDIKRGKFNLQEEQTIIQLHALLGNRWSAIATHLPKRTDNEIKNYWNTHLKKRLVKMGIDPVTHKPKNETPLSSLGLSKNAAILSHTAQWESARLEAEARLARESKLLHLQHYQTKTSSQPHHHHGFTHKSLLPNWTTKPHEDQQQLESPTSTVSFSEMKESIPAKIEFVGSSTGVTLMKEPEHDWINSTMHEFETTQMGEGIEEGFTGLLLGGDSIDRSFSGDKNETAGESSGGDCNYYEDNKNYLDSIFNFVDPSPSDSPMF

>AtMYB106(NP186763)

MPIHVRDREKGRLQNLNRDIFCCVSPSIYQSDAKRAAFVIILIMIISPCCDKAGLKKGPWTPEEDQKLLAYIEEHGHGSWRSLPEKAGLQRCGKSCRLRWTNYLRPDIKRGKFTVQEEQTIIQLHALLGNRWSAIATHLPKRTDNEIKNYWNTHLKKRLIKMGIDPVTHKHKNETLSSSTGQSKNAATLSHMAQWESARLEAEARLARESKLLHLQHYQNNNNLNKSAAPQQHCFTQKTSTNWTKPNQGNGDQQLESPTSTVTFSENLLMPLGIPTDSSRNRNNNNNESSAMIELAVSSSTSSDVSLVKEHEHDWIRQINCGSGGIGEGFTSLLIGDSVGRGLPTGKNEATAGVGNESEYNYYEDNKNYWNSILNLVDSSPSDSATMF

>AmMYBML2(AAV70655)

MGRSPCCDKVGLKKGPWTPEEDQKLLAYIEEHGHGSWRALPARAGLQRCGKSCRLRWTNYLRPDIKRGKISLQEEQTIIQLHALLGNRWSAIATHLPKRTDNEIKNYWNTHLKKRLAKMGIDPVTHKPKSDTLMSNDGQSKNAANLSHMAQWESARLEAEARLVRQSKLQPPSANSFQASTSNPVQKSMGQARCLDVLKAWNGVVWGKNDEAAGVSVTVTTGIVGELGSPTSTLSSAAGTNGIMKEESEEEWKQMPENRDEIGNATPFTSNWESEQVALNSSEACSSREFRGKLYGLVTECFLCRWRGMRIVGYTNSGGNDVGVSDYYEDNKNYGNSILNLVNSSPSHSPIF

>AmMYBML3(AAU13905)

MGRSPFCDKTGLKRGPWTPEEDQKLLACIQEHGHGNWRALPAKAGLERCGKSCRLRWNNYLRPDIKRGKFSSQEEQTIIQLHALLGNRWSAIATHLSRRTDNEIKNYWNTHIKKKLAKMGIDPVTHKPQRDHALSSNNAHVQSKNAANMSHLAQWESARLEAEARLARQSKLQANSECGGGSDTGGGGSDCCGDNKNYWDNILDLVNFSPSDSPIFRVF

>AmMYBML1(CAB43399)

MGRSPCCDKVSLKRGPWTPEEDQKLLSYIQEHGHGSWRALPSKAGLQRCGKSCRLRWSNYLRPDIKRGKFSLQEEQAIIQLHAFLGNRWSAIATHLPKRTDNEIKNYWNTHLKKRLTKMGIDPMTHKPKSHDVLGCGQPKVVANLSHMAQWESARLQAEARLVRESRLVSHHYHSQLLNRATAITHPTLPPCLDVLKVWHGAWTTRPGKDIITSAMFNGFFASNNGNLESPTSILKSSDNMLNASTSVGLLHENPFITDMSYVGKPSNVYEDWVKGIMDNSNELNKIIEPIDSTHYVAHDDDSIGFPGFMEGSTNLVTSTVRTNGPDDDNVVGVFEENDINYWRNVLNVVNSPMGSPVF

>AmMYBMIXTA(CAA55725)

MVRSPCCDKVGVKKGPWTVDEDQKLLAYIEEHGHGSWRSLPLKAGLQRCGKSCRLRWANYLRPDIKRGPFSLQEEQTIIQLHALLGNRWSAIASHLPKRTDNEIKNYWNTHLKKRLTRMGIDPVTHKPHTHNILGHGQPKDVANLNHIAQWESARLQAERRLVRESRLAQNNNKIGTIQRRLTWPLCLDNEQSNHYHSALLNSTSAVGLNQDNSFTNYSARPDNNNIYDDYEVNNIMGMIEFNNNSNYFADSLRLPGFVEGITDISSSNIVLGAGVLPSNSDNVVGYFEENWSSVLNNVASSSSMDSPDVLVNASSSYKMY

>AtMYB17(NP191684)

MGRTPCCDKIGLKKGPWTPEEDEVLVAHIKKNGHGSWRTLPKLAGLLRCGKSCRLRWTNYLRPDIKRGPFTADEEKLVIQLHAILGNRWAAIAAQLPGRTDNEIKNLWNTHLKKRLLSMGLDPRTHEPLPSYGLAKQAPSSPTTRHMAQWESARVEAEARLSRESMLFSPSFYSGVVKTECDHFLRIWNSEIGEAFRNLAPLDESTITSQSPCSRATSTSSALLKSSTNSWGGKEVTVAIHGSDYSPYSNDLEDDSTDSALQLLLDFPISDDDMSFLEENIDSYSQAPPIGLVSMVSKF

>AtMYB3R4(NP_568249)

MEAESSTPQERIPKLRHGRTSGPARRSTRGQWTAEEDEILRKAVHSFKGKNWKKIAEYFKDRTDVQCLHRWQKVLNPELVKGPWTKEEDEMIVQLIEKYGPKKWSTIARFLPGRIGKQCRERWHNHLNPAINKEAWTQEEELLLIRAHQIYGNRWAELTKFLPGRSDNGIKNHWHSSVKKKLDSYMSSGLLDQYQAMPLAPYERSSTLQSTFMQSNIDGNGCLNGQAENEIDSRQNSSMVGCSLSARDFQNGTINIGHDFHPCGNSQENEQTAYHSEQFYYPELEDISVSISEVSYDMEDCSQFPDHNVSTSPSQDYQFDFQELSDISLEMRHNMSEIPMPYTKESKESTLGAPNSTLNIDVATYTNSANVLTPETECCRVLFPDQESEGHSVSRSLTQEPNEFNQVDRRDPILYSSASDRQISEATKSPTQSSSSRFTATAASGKGTLRPAPLIISPDKYSKKSSGLICHPFEVEPKCTTNGNGSFICIGDPSSSTCVDEGTNNSSEEDQSYHVNDPKKLVPVNDFASLAEDRPHSLPKHEPNMTNEQHHEDMGASSSLGFPSFDLPVFNCDLLQSKNDPLHDYSPLGIRKLLMSTMTCMSPLRLWESPTGKKTLVGAQSILRKRTRDLLTPLSEKRSDKKLEIDIAASLAKDFSRLDVMFDETENRQSNFGNSTGVIHGDRENHFHILNGDGEEWSGKPSSLFSHRMPEETMHIRKSLEKVDQICMEANVREKDDSEQDVENVEFFSGILSEHNTGKPVLSTPGQSVTKAEKAQVSTPRNQLQRTLMATSNKEHHSPSSVCLVINSPSRARNKEGHLVDNGTSNENFSIFCGTPFRRGLESPSAWKSPFYINSLLPSPRFDTDLTIEDMGYIFSPGERSYESIGVMTQINEHTSAFAAFADAMEVSISPTNDDARQKKELDKENNDPLLAERRVLDFNDCESPIKATEEVSSYLLKGCR

>PttMYBR3(CAD98760)

MDELKIEERCLENKQLTPASSSSQSEEGSGSAILKSPGVYSPATVSPTNRRTTGPIRRAKGGWTPEEDETLRTAVATYRGKSWKKIAEFFPDRSEVQCLHRWQKVLDPELVKGPWTQEEDDKIVELVAKYGPTKWSVIAKALPGRIGKQCRERWHNHLNPDIKKDAWTLEEELALMNAHRIYGNKWAEIAKVLPGRTDNSIKNHWNSSLKKKLDFYLSTGKLPPISKNGFQNGTKDTNKSAVMKTMKESDSAAQTSSGTTDICKLDEDGKDQLESTLVLDVAASSSALPNEYADSVDLRCCDSELQQKFEDHQDKGIGSGLQFDRSTYGSLYYEPPLVEQLDSYPSNTGSVQHENNSCPVSSPIIFFTPPCVKSRDLSAQSPESILRIAAMSFPNTPSIFRKRKTAQVDLLPSKIGKAGEETVKDRLDMSVEHEKIENTLEKTAAQDGSLCESPASLGNGTIRPNDKPFNASPPYRLRSKRTAVFKSVERQLEFTFEKGRSDGTKPTRLSVKGGSPVTEDCSRATKMGVT
