# Supplementary material for: Members of the MYBMIXTA-like transcription factors may orchestrate the initiation of fiber development in cotton seeds
Source: Front Plant Sci. 2014 May 1;5:179. doi: 10.3389/fpls.2014.00179 (PMC4028877; doi:10.3389/fpls.2014.00179)
Supplement: Supplementary data 3 — Transient assays. [file DataSheet3.DOC]

**Supplemental data 3 (for Figure 1G). Transactivation assay in cotton protoplasts.**

Protoplasts have been isolated from cotyledons of 7-day-old green seedlings of *G. hirsutum* 315-11, grown *in vitro*, cut into 0.5 mm strips and immersed into the enzyme solution for 14-16 hours under vacuum in dark. The enzyme solution is adapted from He et *al*. (2007) with 2% sucrose and with 35% less mannitol/KCl/MES/CellulaseR10/MacerozymeR10.

The effector-reporter assays presented here used a modified version of the pART7 vector (Gleave, 1997). The restriction enzyme sites *Bgl*II and *Sal*I have been introduced in 5’ of the *35S* promoter to allow for the promoter cloning, leading to the vector pART7-FB. The *luciferase* gene from the pDO432 (Ow et al., 1986) vector has been inserted into the multiple cloning site of the pART7-FB using the *Bam*HI site, producing the vector pART7-FB-LUC. The promoter of *MYB25* (BAC125K22, Machado et al., 2009) has been cloned into pART7-FB-LUC by replacing the *35S* promoter; briefly the following primers (5’-3’) GTTTAGATCTGTGGTTTTATGAGGAGTTAATATTGAG (*Bgl*II site, forward primer) and ACCTCTCGAGATATATATGTGTGTGTATGTATGTATCTTTC (*Xho*I site, reverse primer) were used to PCR amplify the *ProMYB25* using the HotStart polymerase (Qiagen), then gel extracted and ligated to pART7-FB-LUC, and sequenced. The effectors *GhMYB25-Like* and *HD-1* coding sequences were respectively PCR amplified from *G. hirsutum* cDNA (Walford et al., 2011, 2012) with the following primers TTCACTCGAGATGCAGCAGTCTCCATGTA (*Xho*I, forward primer) and ACAAGGATCCTCAAAAGACAGAAGAACCAGA (*Bam*HI, reverse primer), and TCGACTCGAGATGTTTAGCCCCAACTTATTTGAGAGT (*Xho*I site, forward primer) and TCGAGAATTCTCAAGCATTATTGCACATTACGGCA (*Eco*RI, reverse primer), then gel extracted and ligated to pART7-FB (see sequences below).

To normalise the luciferase assay we used the pART7-FB carrying the *beta galactosidase* gene as a calibrator. The *beta galactosidase* has been introduced into pART7-FB using the primers TCGAAAGCTTATGTCGTTTACTTTGACCAACAAG (HindIII, forward) and TCGATCTAGATTTTTGACACCAGACCAACTGG (XbaI, reverse) through the steps of PCR amplification based on the pEF-ENTR-Lac (provided by Adgene Cat#17430, Campeau et al., 2009), digestion, purification, ligation and sequencing.

The transfection of protoplasts was performed using high quality plasmid DNA isolated form bacteria with the JETstar 2.0 Plasmid Maxiprep kit (Astral, Cat#220020). Twenty μg of effector(s) and reporter plasmid DNA, and 30μg of calibrator were transfected together according to He et al. (2007). After transfection the protoplasts were incubated 48 hours at room temperature under low light intensity then collected as described in He et al. (2007) and immediately frozen in liquid nitrogen. After lysis of the protoplasts the extract has been immediately used for Luciferase emission, measured by mixing 100μL of extract to 100μL Britelite plus (Perkin Elmer, Cat#6016761) and analysed in a plate reader Wallac 1420 (Perkin Elmer). Beta galactosidase assay was also carried out in a in a plate reader. The level of reporter activation by the effector(s) was calculating as follow: LUCIFERASE activity (CPS, counts per second) / β GALACTOSIDASE activity (OD_414nm_).

**Campeau E, Ruhl V, Rodier F, Smith C, Rahmberg B, FUSS J, *et al***. (2009) A versatile viral system for expression and depletion of proteins in mammalian cells. PLoS ONE. **4**, e6529.

**Gleave AP** (1992). A versatile binary vector system with a T-DNA organisational structure conducive to efficient integration of cloned DNA into the plant genome. Plant Mol Biol **20**, 1203-1207.

**He P, Shan L, and Sheen J** (2007) The use of protoplasts to study innate immune responses. Methods in Molecular Biology, vol. 354: Plant–Pathogen Interactions: Methods and Protocols Edited by: P. C. Ronald © Humana Press Inc., Totowa, NJ

**Machado, A., Wu, Y., Yang, Y., Llewellyn, D.J. and Dennis, E.S.** (2009). The MYB transcription factor *GhMYB25* regulates early fibre and trichome development. The Plant J. **59**, 52-62.

**Ow DA, Wood KV, Deluca M, De Wet JR, Helsinki DR and Howellt SH** (1986) Transient and Stable Expression of the Firefly Luciferase Gene in Plant Cells and Transgenic Plants. Science **234**, 856-859

**Walford, S.A., Wu, Y.R., Llewellyn, D.J., and Dennis, E.S.** (2011). *GhMYB25-like:* a key factor in early cotton fibre development*.* The Plant J. **65,** 785-797.

**Walford, S.A., Y. Wu, D.J. Llewellyn, and Dennis,** **E.S.** (2012). Epidermal cell differentiation in cotton mediated by the homeodomain leucine zipper gene, *GhHD-1*. Plant J. **71**, 464-78.

Sequences used in this study (5’to3’):

>GhMyb25Promoter

GTGGTTTTATGAGGAGTTAATATTGAGCTTGTAGCTGCTGATCAACTCTATTTAATGGTAGTGATTCCAATCCAAAATCCGCCTCAATTTTGGATGGATTGTCGGGCACTTGATTTCAAAGGGGTAGAAAAGCTAATTCATAATTTCTTCATTTTTCTCCTAATAAATATTTCCAAAATCTAAAGCCAAGGTACCTCTCTCCGGGATATTAGATTATGTTAAGCATGGGGTGAGTATAGGCTTAGATGATTATTATGGAAGACATAAATATCACAAATGCTTCAACCTAACAGCAAAGCTAGGAATTAAATCACCTTTTTTTTTTCTTACCACGTTTTTCAGAAATTGGGGGTAATTTCCACAATTTCTGCAAACTTAATCATTTATTACAAGTAGCTCCACCATTTTATTTCATCTTAAATGAAGAATTCATAAAAAAATCTTTGCTTCATCAAATTTCTCAACAGCTTATAGATTAACATTGCTATCATTATTAATAAAATCTCATTAATGTAATTAATTAATCTGATTTAATGATTAATTACTTGAAAACTTTCAAGGAAAAAAAACATTTAAGTGCCATTAAAATGTATGATTAATGAAACGAAGAAACAAAGATTTTATTAATACACCATTTCTTTGTTATATATGTATACATACCTTGAATTAATTCGTAGCATTTTTTACTTAATTATTAATTCTTTCTTTTTAAAATCAAATGTTTGGTCGAATTGTTGTTGTTTTGAAACCATGCTTTTAATTAATCACTTAATAATTACATTTTAAAGCAAATGTATATCCAATTTACACATACAACCATCCTCTCCTTTCTTTTTTTTATTTTTTGGGAAATGAAAAACCACCGACTTTCTCCCAAAGTCTTAGGAACAAAAAAAAAAAATCTTTTTATATTAAATGCCACCTATTAAAACATGGTCATGATATCAAATTAAAATTTTCAACCTATATCAAATACACCCTAAATCTCAAATTAATTTTTATATGCTTACAATCATATATCATGATGGTTGTGATTCTTTTTCTTTAAAACAACCTTATTATATTAATTGGTATTTGAGTTTAATATTTTTTTTTAATGTGGTACTTATATTTTGTAAAAATTATACATTTTGGTACCCAAAGATTATAATGTTGACTTGTTACTTTTTATTGTGTAATTCGAGTTTTAAAGTTGATTTGATTAAAAGTAATTGCTAATAATATTAGGTTTGAATTCTCATTATTTTTTTAATAGAGTAAATTTAGTGTTAATTGTGAATTCATTTAATTAATTAAATTGATGACCGTGAATTTTATCAATTTTTAGGTTTGATTTTAATAATAGATTAAGGGTATTGATGGTATCATGTATGCAAAAATGAGTTAGAAAAAAATTGTGAATCTGATCAAGTAATAAATATATATAAAGAAAATTAAATGACATTGGTTGTCCATAATAAATTGATTAACTATATCTAGATGTAATAATAAAGGAGGGTATAATGGTCATATATCATTATCTATGTTTACAACACAATTGCCTTTATATTATATCGCACTCATATCCCTACTCCCAATCACCTCTCCCCTCATTTTCATCGCTTTCAACTTTATATATATGTTCATTGTGTTGATCCAAAAAGCCAAAGGGAGTTCACAGAAATTGTTATACAGTTCTAGCTAAGGTTCATTTGAAAGATACATACATACACACACATATATAT

>GhHD-1 coding region

atgtttagccccaacttatttgagagtccccatatgttcgacatgtctcataagacctcggaaagtgaactaatggggaaggtcagggatgatgattatgagatcaaatcagtcactgaaactatggatgctccctctggagatgatcaagatcctgaccaacgccctaaaatgaagtgttatcatcgtcatacccagcgtcaaatccaggagatggaagcattctttaaggagtgccctcaccctgatgataagcaaaggaaggagcttggccgtgagctagggttagaacctcttcaagtcaagttttggttccaaaacaagcgcacccaaatgaaggcccaacatgaacgccatgaaaatgctatactgaaggctgagaatgaaaaactccgagccgagaataataggtacaaggaagctctcagcaatgctacatgccccagctgtggaggcccagctgcccttggagagatgtcatttgatgagcaacttttgagaatagaaaatgctcggttaagggaagagattgataggatatctggaatagctgctaaatatgttggcaagcctttatcttctttgcctcacctttcatctcatttacattcgcgctctgttgatcttggagctagcaatttcgggacacaatcaggatttgtaggggaaatggatcgcagtggtgatcttctgaggtctgtctctggacctacagaagcggataagcccatgattgttgagcttgctgttgctgcaatggaggaactaatacgaatggcccaatctggggaacctttgtgggttcctggggacaattctatagatgtgttgagcgaagatgaatacttaagaactttccctaggggaattggaccaaagcctttggggttgaggtctgaagcttcaagagaatctgcagttgtcatcatgaatcatgtcaacttagttgagattctcatggatgtgaatcaatggtcaagtgtgttttgcggtattgtttcaagggctatgactttagaagtcctatcaactggagttgcaggaaactacaatggagccttgcaagtgatgacggctgagttccaagtcccttcaccacttgtaccaactcgggaaaattatttcgtgaggtactgtaagcagcatattgatggaacttgggcagtggttgatgtttccttggataatttacgccctaacccaatgtccaagtgtagaagaaggccctcaggttgcttgatccaagaattgccaaatggatactctaaggttatatgggtcgagcatgtagaagtggatgatagagctatccacaacatatacagaccagtagttaattccggtctagcttttggagcaaaacgttgggtggctacgttagatcgacagtgtgagcgtctagcaagttcaatggccagtaacattccggcaggggatctatgcgttataacaagcctagaagggaggaaaagtatgttgaagttggcagagaggatggtgactagcttttgtacaggtgttggtgcttctacggcccatgcttggacaagtttatcggcaacaggctccgatgatgtgcgggttatgacccgaaagagcatggatgatccaggaaggcctcctggtattgtacttagtgctgcaacttccttctggatcccagttccaccaaagagggtatttgatttcctaagggatgagaactctagaagtgagtgggatatcctatcaaatggtggcctagttcaagaaatggctcacatagctaatggtcgtgatccaggcaattgtgtctctttactgcgcgtaaatagtgcaaactctagccaaagcaacatgttgatacttcaagagagctgcactgatgctacagggtcctatgtgatatatgccccggtcgatattgttgcaatgaacgtcgtcttaagtgggggggacccggattatctcgcactattgccatccggtttcgcaattctacccgatggtccaggagttaatggaggagggattctcgaaataggctcgggtggctctctccttaccgttgctttccagattttggttgattcagttccaacagcaaagctttcccttggatcagtgacgactgtcaatagtctaattaaatgcacagttgaaaggatcaaggctgccgtaatgtgcaataatgcttga

>GhMYB25-Like coding region

ATGCAGCAGTCTCCATGTAGCGACAAGGTGGGGTTGAAGAAAGGGCCATGGACTCCAGAAGAAGACCAAAAACTCTTGTCTTATATTCAAGAACACGGCGGTGGAAGCTGGCGAGGCTTGCCCGCAAAAGCTGGACTTCAAAGATGTGGCAAGAGTTGTAGACTTAGGTGGATTAACTACTTAAGACCAGATATCAAAAGAGGAAAGTTCAGTTCGCAGGAAGAACGAACCATCATTCAACTCCACGCCCTTCTTGGAAACAGGTGGTCGGCTATTGCGGCTCATTTGCCAAAAAGAACAGACAATGAGATCAAGAACTACTGGAATACACAGTTGAAGAAAAGGTTGACGACGATAGGGATCGACCCTGCAACTCACAGGCCTAAAACCGATACCCTCGGTTCAACTCCCAAGGATGCCGCTAACCTTAGCCACATGGCTCAATGGGAGAGTGCTCGGTTAGAAGCTGAAGCTAGATTGGTGAGAGAGTCGAAACGAGTTTCAAACCCTTCGCAAAACCAATTTAGGTTCACGTCTTCATCGGCTCCTCCACTGGTAAGCAAAATTGATGTTGGTTTGGCTCATGCTACTAAACCGCAATGCCTCGATGTACTCAAAGCTTGGCAACGTGTAGTCACTGGATTGTTCACTTTCAACACTGACAACCTCCAATCTCCAACATCGACGTCGAGCTTCACGGAAAACACGTTACCAATCTCATCTGTCGGGTTCATTGACAGCTTTGTGGGGAACTCAAATAACAGCTGTTGCGGAAATAATTGGGAATGTGTGGAGAAATCGAGCCAAGTTGCTGAATTACAGGAAAGATTGGATAACTCAATGGGGTTGCATGACATATTGGATCTCTCCTCAGAAGATGTATGGTTTCAAGGCTCATACAGGGCGGAAAATATGATGGAAGGGTATTCGGACACGTTAATGGTTTGTGATTCTGGGGATCATCCGAAGAGTTTGTCAATGGAGCCTAGACAAAACTTTAATGTTGGAACAAGTAATGCTAGTAGTTTCGAAGAAAACAAGAATTACTGGAACAACATCCTTAATTTTGCGAATGCTTCCCCTTCTGGTTCTTCTGTCTTTTGA
